# Dynamic neural fields as a step toward cognitive neuromorphic architectures

**DOI:** 10.3389/fnins.2013.00276

**Published:** 2014-01-22

**Authors:** Yulia Sandamirskaya

**Affiliations:** Chair for Theory of Cognitive Systems, Institute for Neural Computation, Ruhr-University BochumBochum, Germany

**Keywords:** dynamic neural fields, cognitive neuromorphic architecture, soft winner-take-all, autonomous learning, neural dynamics

## Abstract

Dynamic Field Theory (DFT) is an established framework for modeling embodied cognition. In DFT, elementary cognitive functions such as memory formation, formation of grounded representations, attentional processes, decision making, adaptation, and learning emerge from neuronal dynamics. The basic computational element of this framework is a Dynamic Neural Field (DNF). Under constraints on the time-scale of the dynamics, the DNF is computationally equivalent to a soft winner-take-all (WTA) network, which is considered one of the basic computational units in neuronal processing. Recently, it has been shown how a WTA network may be implemented in neuromorphic hardware, such as analog Very Large Scale Integration (VLSI) device. This paper leverages the relationship between DFT and soft WTA networks to systematically revise and integrate established DFT mechanisms that have previously been spread among different architectures. In addition, I also identify some novel computational and architectural mechanisms of DFT which may be implemented in neuromorphic VLSI devices using WTA networks as an intermediate computational layer. These specific mechanisms include the stabilization of working memory, the coupling of sensory systems to motor dynamics, intentionality, and autonomous learning. I further demonstrate how all these elements may be integrated into a unified architecture to generate behavior and autonomous learning.

## 1. Introduction

Organisms, such as animals and humans, are remarkable in their ability to generate behavior in complex and changing environments. Their neural systems solve challenging problems of perception and movement generation in the real world with a flexibility, adaptability, and robustness that surpasses the capabilities of any technical system available today. The question of how biological neural systems cope with the complexity and dynamics of real-world environments and achieve their behavioral goals, does not have a simple answer. Processes such as memory formation, attention, adaptation, and learning all play crucial roles in the biological solution to the problem of behavior generation in real-world environments. Understanding how these processes are realized by the neural networks of biological brains is at the core of understanding biological cognition and building cognitive artifacts that successfully contend with real world constraints.

The field of neuromorphic engineering may contribute to the ambitious goal of understanding these cognitive processes by offering platforms in which neural models may be implemented in hardware using the VLSI (Very Large Scale Integration) technology. The analog neuromorphic hardware shares several properties with biological neural networks such as the presence of the inherent noise, the potential mismatch of computing elements, constraints on connectivity, and a limited number of learning mechanisms. Apart from these shared constraints, artificial and biological neural networks also maintain the advantages of pervasive parallel computation, redundant systems to handle sensory and motor noise, and low power consumption. Success in the implementation of cognitive models on neuromorphic hardware may lead to breakthroughs both in understanding the neural basis of human cognition and in the development of performant technical systems (robots) acting in real-world environments.

VLSI technology allows one to implement large neural networks in hardware by configuring the VLSI device to simulate the dynamics and connectivity of a network of spiking neurons. Such networks may be efficiently configured, connected to sensors and motors, and operate in real time (Mead and Ismail, 1989; Indiveri et al., 2009, 2011). However, a challenging question remains: how to develop these neuromorphic systems beyond simple feed-forward reactive architectures toward architectures capable of cognitive behavior?

Soft winner-take-all (WTA) connectivity has been recently proposed as an important milestone on the way toward such functional cognitive neuromorphic systems (Indiveri et al., 2009; Rutishauser and Douglas, 2009). Soft WTA networks are computational elements that are hypothesized to play a central role in cortical processing (Douglas and Martin, 2004; Rutishauser and Douglas, 2009). Recently, a wide variety of WTA networks of spiking neurons have been implemented in hardware (Indiveri et al., 2001; Abrahamsen et al., 2004; Oster and Liu, 2004; Indiveri et al., 2009). These initial architectures have made use of WTA connectivity to enable the effective processing of sensory information (Liu and Delbruck, 2010) and the implementation of finite state machines (Neftci et al., 2013). Soft WTAs introduce a *cognitive* layer to the neuromorphic hardware systems, which enables reliable processing on unreliable elements (Neftci et al., 2013). The WTA networks contribute to making neuromorphic systems more cognitive, because they stabilize localized attractor patterns in neural networks. These stable attractors organize the dynamics of the neural system in a macroscopical way and enable the coupling of the network to sensors and motors despite noise, fluctuations, and neural mismatch. WTA connectivity therefore introduces macroscopic neural dynamic states which may persist long enough to interact with other parts of the neural-dynamic architecture, thus moving neuromorphic systems beyond mere reactive behavior.

However, there are still open questions on the way toward cognitive processing with hardware WTAs. The first question concerns representational power: How can we add contents to the state in a WTA network and link this network state to perceptual or motor variables? How can the system represent associations and concepts such as “a red ball on the table” or “a hand moving toward an object” in this framework? The second line of open questions concerns movement generation and the motor behavior: How should the system represent and control movements in this framework? How should it decide when to initiate or terminate a movement? Finally, questions regarding learning also arise: How may a system learn WTA connectivity of its neural network? How may the system learn the connections between WTA networks in a complex architecture? Such questions are often addressed in the fields of psychophysics, cognitive science, and artificial intelligence, but the proposed models and solutions are often not compatible with neural implementations. Here, I propose that Dynamic Field Theory (DFT) is a framework which may make such cognitive models feasible for neuromorphic implementation because it formulates the principles of cognitive representations and processes in a language compatible with neuromorphic soft WTA architectures. Identifying the computational and architectural principles underlying these cognitive models may facilitate the development of large-scale neuromorphic cognitive systems.

DFT is a mathematical and conceptual framework which was developed to model embodied human cognition (Schoner, 2008). DFT is an established framework in modeling many aspects of human cognition and development including visual and spatial working memory, object and scene representation, sequence generation, and spatial language (Johnson et al., 2008). DFT cognitive models have been used to control robots and demonstrate that the developed architectures can function autonomously in the real-world (Erlhagen and Bicho, 2006; Sandamirskaya et al., 2013). DFT builds on Dynamic Neural Fields (DNFs), which, as I will discuss in the Methods section, are analogous to soft WTAs in their dynamics and lateral connectivity within networks (Neftci et al., 2010). Accordingly, their dynamical and structural principles may be applied to the design of neuromorphic WTA architectures.

In this paper, I discuss computational and architectural principles recently developed in DFT that may be applied to WTA neuromorphic networks. These principles can increase the representational power and autonomy of such networks, and thus contribute to the greater scalability and robustness of neuromorphic architectures. In particular, these principles enable the coupling of DNFs of differing dimensionality, the coupling of the architectures to sensors and motors, cognitive control over behavior, and autonomous learning. On a simple exemplar architecture, I demonstrate how these principles enable autonomous behavior and learning in a neural-dynamic system coupled to real-world sensors and motors. I also discuss the possibility of implementing DNF architectures in neuromorphic hardware.

## 2. Materials and methods

### 2.1. Dynamic neural fields: basic dynamics and instabilities

A DNF is a mathematical description of activation dynamics of a neuronal population in response to certain parameters of the agent's behavioral state. The behavioral parameters, such as a perceptual feature, location, or motor control variable, span dimension(s), over which the DNFs are defined (Schoner, 2008). The dynamics of DNF may be mathematically formalized as a differential equation, Equations (1–3), which was first analyzed by Amari (1977), and used to model neuronal dynamics on a population level (Wilson and Cowan, 1973; Grossberg, 1988; Ermentrout, 1998).

(1) $$\tau \dot{u}(x,t)=-u(x,t)+h+\text{​​}\int \text{​}\text{​}f(u(x^{\prime },t))\omega (x-x^{\prime })dx^{\prime }+S(x,t),$$

(2) $$\omega (x-x^{\prime })=c_{exc}\exp\left[-\frac{{\left(x-x^{\prime }\right)}^{2}}{2\sigma_{exc}^{2}}\right]-c_{inh}\exp\left[-\frac{{\left(x-x^{\prime }\right)}^{2}}{2\sigma_{inh}^{2}}\right]\text{​},$$

(3) $$f(u(x,t))=\frac{1}{1+\exp[-\beta u(x,t)]}.$$

In Equation (1), *u*(*x, t*) is the activation of the DNF over dimension *x*, to which the underlying neuronal population is responsive. *h* is a negative resting level and *S*(*x, t*) is an external input driving the DNF. The lateral interactions in DFT are shaped by a symmetrical homogeneous interaction kernel, Equation (2), with a short-range excitation and a long-range inhibition (Ellias and Grossberg, 1975); σ_*exc*_, σ_*inh*_, *c*_*exc*_, and *c*_*inh*_ are the width and the amplitude of the excitatory and the inhibitory parts of the interaction kernel respectively. The sigmoidal non-linearity, Equation (3), shapes the output of the DNF in such a way, that only sufficiently activated field locations contribute to neural interactions; β determines the slope of the sigmoid.

**Figure 1:**
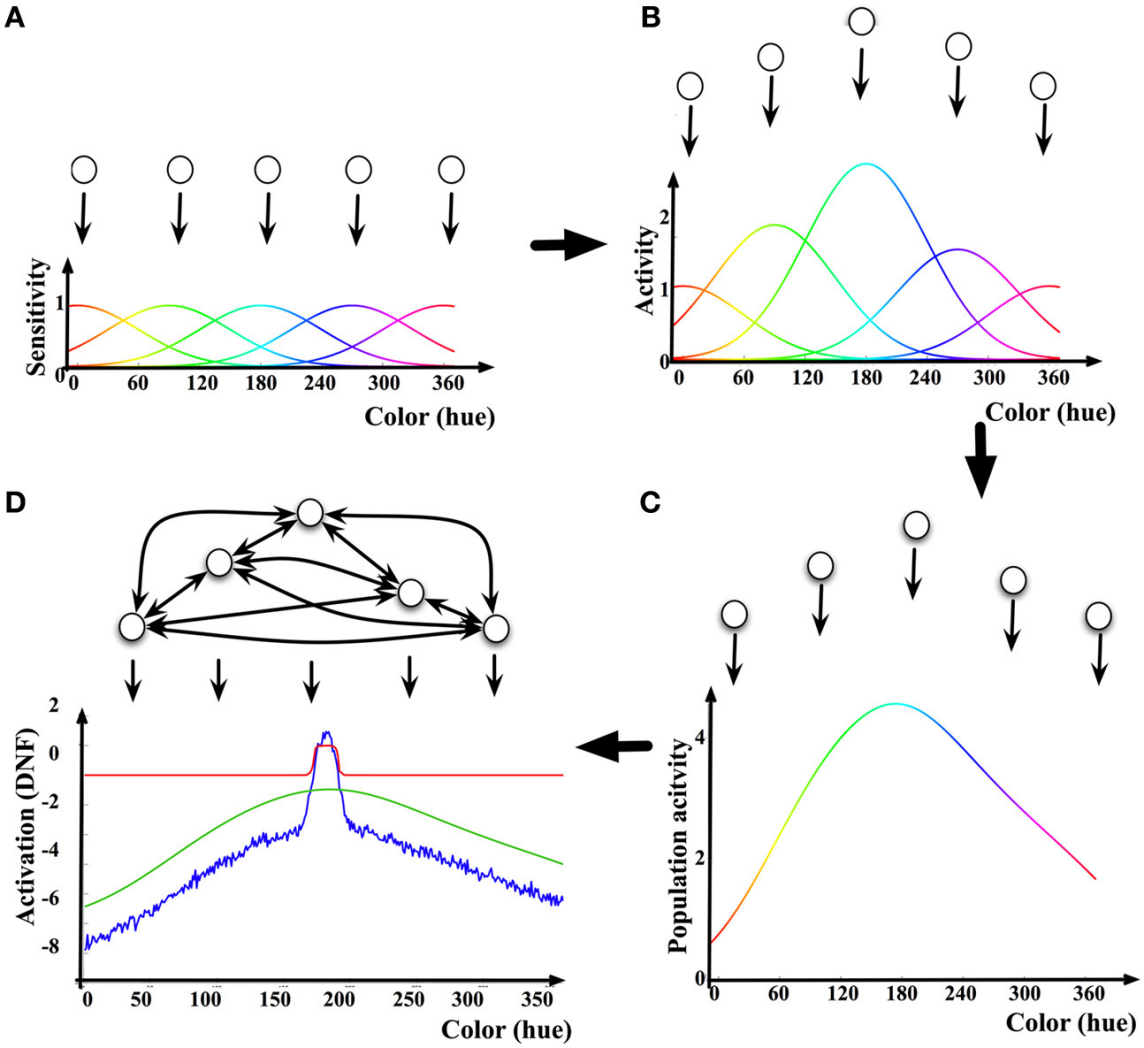
**Illustration of the relationship between neuronal activity and a DNF**. **(A)** Five exemplar “neurons” (neuronal populations) and their tuning curves in the color dimension. **(B)** The tuning curves are scaled by the mean firing rate (activation) of the neurons. **(C)** By summing the scaled tuning curves, the Dynamic Population Activity [DPA, Bastian et al. (2003)] curve in response to a given color stimulus is constructed. **(D)** The DNF dynamics adds lateral interactions between neurons according to Equation (1). The activation of the DNF is shown as a blue line, the red line shows the output (sigmoided activation) of the DNF, the green line is the DPA [same as in **(C)**].

An example of how a DNF may be linked to the activity of a neuronal population is shown in Figure 1: First, each neuron in the population contributes its tuning curve in respect to the behavioral parameter of interest as a (virtual) input to the DNF. The tuning curve is determined as a dependence of the mean firing rate or the action potential of the neuron on the value of the behavioral parameter (Figure 1A). Second, the tuning curves of the neurons in the population are summed, each weighted by the current activation level (e.g., mean firing rate) of the respective neuron. The resulting Distribution of Population Activity [DPA, introduced by Bastian et al. (2003) to derive a DNF description of neuronal data on movement preparation in studies of reaching movements in monkeys] represents the overall activity of the selected neuronal population in response to a given stimulus or state of the behaving neural system (Figures 1B,C). Finally, the neurons in the population are assumed to be interconnected so that the nearby (in the behavioral space) locations exert excitatory influence on each other, and the far-off locations inhibit each other (“on-center, off-surround” connectivity Ellias and Grossberg, 1975). The resulting activation function *u*(*x, t*), is activation of the DNF. A sigmoidal non-linearity *f*(*u*(*x, t*)), shapes the output of the DNF, which impacts on the DNF itself through the lateral connections and on the other parts of the neural architecture connected to this DNF.

The pattern of lateral connectivity of DNFs results in existence of a localized-bump solution in their dynamics (Figure 1D), which is at the core of the properties of DNFs to exert elementary cognitive functions, discussed further. In the realm of modeling human cognition, activity peaks bridge the low-level, graded sensory-motor representations to categorical, symbol-like representations. The localized (and stabilized, i.e., sustainable over macroscopical time intervals) representation facilitates perception, action generation, and learning.

The connectivity pattern within DNF also makes it a soft WTA architecture. Indeed, a WTA-connected network may be formalized in terms of two neuronal populations, an excitatory and an inhibitory one (Rutishauser and Douglas, 2009):

(4) $$\tau \dot{x_{i}}=-x_{i}+f(I_{i}+\alpha x_{i}-\beta_{1}x_{N}-T_{i})$$

(5) $$\tau \dot{x_{N}}=-x_{N}+f(\beta_{2}\sum_{j=1}^{N-1}x_{j}-T_{N}).$$

In Equations (4, 5), the excitatory population of nodes (neurons) *x*_*i*_ has an attractor dynamics driven by the external input, *I*_*i*_, the resting level potential, *T*_*i*_, the self-excitatory term with strength α, and the inhibitory term with strength β_*1*_. The inhibition is shared by all excitatory nodes and is provided by the inhibitory neuron, *x*_*N*_, which also follows an attractor dynamics, driven by activity in the excitatory population and the resting level *T*_*N*_.

In these equations, the excitation constant, α, is analogous to the excitatory part of the interaction kernel of a DNF, *c*_*exc*_ in Equation (2), and the strength of the coupling of the inhibitory population onto the excitatory population, β_1_, corresponds to the inhibitory part of the interaction kernel with the strength *c*_*inh*_. In the DNF equation, the inhibition is coupled into the field's dynamics without delay, which is present in the WTA network of Equations (4, 5).

In several studies on development of working memory and spatial cognition in infants and toddlers, a more general DNF equation is used, in which a separate inhibitory layer is introduced [e.g., Johnson et al. (2006,2008)]. Separate inhibitory layer leads to a delay in the inhibitory interaction among neural field's locations, which allows to model fine-grained effects in competition among items in the working memory depending on timing of their presentation. The separate inhibitory layer is also used to create a shared inhibition among perceptual and working memory neural fields, which plays a critical role in a change detection process.

When DNF architectures are created to generate behavior in an embodied agent, the DFT postulates that only attractor states impact on the behavior of the controlled agent and thus the dynamics of DNFs is typically tuned to relax as fast as possible to the attractor state. Since this holds for the separate inhibitory layer, the presence of the delay in the inhibitory dynamics is negligible in robotic DNF architectures. For this reason, when DNFs are used to control robots, only single-layer dynamics are used, where inhibition and excitation are integrated in a single equation. Since WTA dynamics in Equations (4,5) is a more general formulation than DNFs, discussed in this paper, the equivalence between these two mathematical structures requires a constraint on the timing constant of the inhibitory population, which needs to be faster than the timing constant of the excitatory population, which in its turn is faster than the dynamics of sensor inputs to the field.

The stable localized activity peak solution of the DNF dynamics is the DNF variant of soft-WTA behavior. Intuitively, the short-range excitatory interactions stabilize the peak solution against decay and the long-range inhibitory interactions stabilize peaks against spread by diffusion. The sites of the DNF, which have above zero activity, are the “winners” of the DNF dynamics. The sigmoidal non-linearity increases stability of the localized peak. The important contribution of DFT to understanding the dynamics of soft WTA networks is the characterization of stable states and instabilities between them based on the analysis of Equation (1) (Amari, 1977; Schoner, 2008; Sandamirskaya et al., 2013):

- The *detection instability* separates a quiescent state of the DNF from an active state. In the quiescent state, the inputs are not strong enough to collectively drive the DNF over the activation threshold. The DNF produces no output in this state, it is invisible for the down-stream structures, driven by the DNF. To the contrary, when inputs are strong enough to drive the field over the activation threshold in one or several locations, an activity peak emerges in the field, which provides input to the down-stream structures, or the motor system.
- The DNF's inputs may drive the field over the threshold at several locations. In this case, the field may build several activation peaks or it may select and amplify activity at one location only, depending on the spread of the lateral inhibition. In the latter case, a *selection instability* separates an inactive state from an activated state of the DNF dynamics.
- If the lateral interactions are strong enough, a peak in the DNF may be sustained even if the input, which initiated the peak, ceases. This *working memory instability* separates the state of the field with no activation from the state, in which an external inhibiting input is needed to deactivate the field.
- A negative external input or a decrease of the excitatory input may lead to an extinction of the activity peak. This causes a *reverse detection instability*, or forgetting instability, which separates an active state from the quiescent state.

The localized-peak stable states and instabilities between them form the basis for more complex DNF architectures, just as WTA networks form the basis for state-based spiking network architectures. In the following, I present additional components in the DFT, which may be translated into VLSI WTA networks and enhance their scalability and autonomy.

### 2.2. Coupling dynamic neural fields to sensory systems

**Figure 2:**
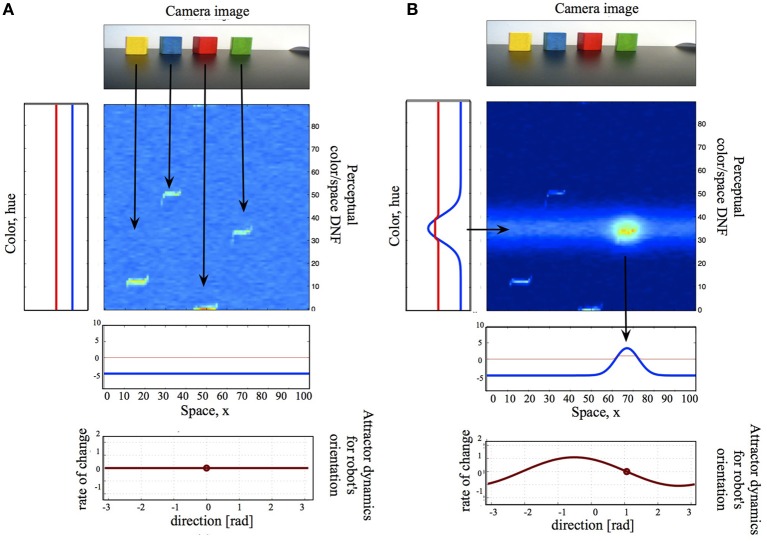
**A small DNF architecture, which consists of a two-dimensional color-space DNF (center), one-dimensional color- and space- DNFs, coupled to the perceptual DNF, a camera input (top), and an attractor motor dynamics (bottom)**. **(A)** The camera input alone is not sufficient to activate the perceptual DNF, the system is quiescent and produces neither output nor behavior. **(B)** A color cue creates an activity peak in the color DNF over the hue value of the named color. This activity peak is projected onto the 2D perceptual DNF as a subthreshold activity ridge, which overlaps with the camera input for the green object. The resulting activity peak in the 2D DNF provides input to the spatial DNF, which, in its turn, sets an attractor for the motor dynamics. The latter drives the motor system of the agent, initiating an overt action.

Figure 2 shows a small DNF architecture, which exemplifies the coupling structures in DFT: coupling the DNFs to each other, to sensors, and to motors. Here, I will introduce the principles behind these coupling structures, while referring to the figure for a concrete example. Overall, the simple system in the Figure 2 performs saliency computations based on color- or spatial cues by means of neuronal dynamics (DNF or WTA computation) and will be a building block, used in the example, presented in Section 3.

In Figure 2, a two-dimensional perceptual color-space DNF receives input from the robotic camera. Camera input to this DNF is constructed in the following way. The raw hue value of every pixel corresponds to the vertical location in the DNF, the location of the pixel on the horizontal axis of the image to the horizontal location in DNF, and the saturation value of the pixel to the intensity value of the sensory input. Thus, the input to the perceptual DNF is an unsegmented stream of color-space associations. If the input is strong enough to pass the activation threshold and is localized in space, a peak of suprathreshold activity evolves, which represents the perceived object. In Figure 2A, the camera input is not sufficient to activate the perceptual DNF—only subthreshold hills of activity represent the four salient objects in the visual scene. However, when the perceptual DNF receives an additional input, which specifies the color of the target object and which overlaps with one of the subthreshold hills, an activity peak evolves in the perceptual DNF and signals the selection of an object of interest (Figure 2B). The additional input arrives from another—color—DNF, which is coupled to the perceptual DNF, as described in Section 2.3.

**Figure 3:**
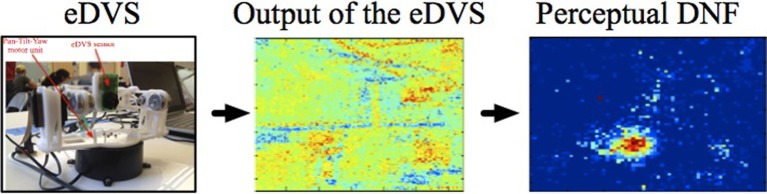
**The neuromorphic Dynamic Vision Sensor [eDVS, Conradt et al. (2009)] on a pan-tilt unit, the output of the eDVS, integrated over a time window of 100 ms, and the instantaneous output of the perceptual DNF**. The perceptual DNF enhances the perceptual input in a selected region (which reached the activation threshold first), and inhibits all other locations in the visual array, performing an elementary object segregation operation.

Another example of coupling a sensor to the DNF is shown in Figure 3. Here, a neuromorphic embedded Dynamic Vision Sensor [eDVS, Conradt et al. (2009)] drives the perceptual DNF. In the eDVS, each pixel sends an event when it sensed luminance changes. Consequently, the sensor naturally detects moving objects. If the object of interest is not moving too fast relative to the motor capabilities of the agent, the perceptual DNF may be used to stabilize the representation of the instantaneous position of the moving object in order to use this position to parametrize the motor action (e.g., to direct the agent's gaze toward the object). If the object is moving too fast for the behaving system, a predictive mechanism needs to be built into the DNF's dynamics (Erlhagen and Bicho, 2006).

### 2.3. Dynamic neural fields of higher dimensionality and couplings

A single DNF describes activation of a neuronal population, which is sensitive to a particular behavioral parameter. Activity of any behaving agent, however, is characterized by many such parameters from different sensory-motor modalities. In DFT, there are two ways to represent such multimodality of a system: multidimensional DNFs and coupled DNFs.

The multidimensional DNFs are sensitive to combinations of two or several behavioral parameters. The perceptual color-space field in Figure 2 is an example of a two-dimensional DNF, which may be activated by combinations of color and locations in space. Such multidimensional DNFs have typically low dimensionality.

Two DNFs of the same or different dimensionality may be coupled with weighted connections, according to Equation (7) (Zibner et al., 2011).

(6) $$\tau \dot{u_{1}}(x,t)=-u_{1}(x,t)+h+\int f(u_{1}(x^{\prime },t))\omega (x-x^{\prime })dx^{\prime }+S(x,t),$$

(7) $$\tau \dot{u_{2}}(y,t)=-u_{2}(y,t)+h+\int f(u_{2}(y^{\prime },t))\omega (y-y^{\prime })dy^{\prime }+W(x,y)\times f(u_{1}(x,t)).$$

Here, *u*_1_(*x, t*) and *u*_2_(*y, t*) are two DNFs, defined over two different behavioral spaces, *x* and *y*. The first DNF provides an additive input to the second DNF through the (adaptable) connection weights matrix, *W*(*x, y*), which maps the dimensions of the space *x* onto dimensions of the space *y*.

For example, the one-dimensional color DNF in Figure 2 represents distributions in the color (hue) dimension. This DNF projects its activation onto the two-dimensional color-space DNF. In particular, since the two DNFs share one dimension (color), the output of the one-dimensional DNF is copied along the not shared dimension (space) of the two-dimensional DNF. This typically results in a ridge-shaped input to the two-dimensional DNF (stamming from the Gaussian shape of the activity peak in the one-dimensional DNF). If this ridge overlaps with a localized subthreshold input in the two-dimensional DNF, an activity peak evolves over the cued (in this case, by color) location (Zibner et al., 2011).

Further, the localized output of the two-dimensional perceptual DNF in Figure 2 is in its turn projected on a one-dimensional spatial DNF, which represents locations on the horizontal axis of the image plane. This projection may be either a sum or a maximum of the DNF's output in the dimension, not shared between the two DNFs (here, color). An example of an adaptive coupling between DNFs of the same dimensionality is presented in Section 2.6.2.

In terms of WTA network, coupling between two DNFs is equivalent (under constraints, stated in Section 2.1) to two WTA networks, one of which receives output from the other one as an external input, which is mapped through synaptic connections.

### 2.4. Coupling the DNF to attractor motor dynamics

In order to close the behavioral loop, DNF architectures have to be coupled to the motor system of a behaving agent. The control of motor actions may be expressed mathematically as an attractor dynamics, where the neural system sets attractors for motor variables, such as position, velocity, or force of the effector. Deviations from the attractor due to an external or an internal perturbation are then actively corrected by the neural controller in the motor system. Such motor attractor dynamics have been probed in control of mobile robots (Bicho and Schoner, 1997) and multi degrees of freedom actuators (Schaal et al., 2003; Iossifidis and Schöner, 2004; Reimann et al., 2011), and also used to model human motor control (Latash et al., 2007).

In order to couple the DNF dynamics to the attractor dynamics for motor control, the space-code representation of the DNF (in terms of locations of activity peaks) has to be mapped onto the rate-code representation of the motor dynamics (in terms of the value of the control variable). Figure 2 (bottom) and Equation (8–9) show how the space-code of a DNF may be translated into the rate-code of attractor dynamics through a weighted projection to the rate-coding neural node. The weights (or gain field, λ(*x*)) of this projection may be subject to learning (or adaptation) (see Section 3).

(8) $$\tau \dot{u}(x,t)=-u(x,t)+h+\int f(u(x^{\prime },t))\omega (x-x^{\prime })dx^{\prime }+S(x,t),$$

(9) $$\tau \dot{\phi }(t)=-\phi \int f(u(x,t))dx+\int \lambda (x)f(u(x,t))dx.$$

Here, *u*(*x, t*) is a one-dimensional motor DNF, which represents the target values of the motor variable using space coding. ϕ is the motor variable, which controls movement of the robot (e.g., velocity, position, force of a motor, or the target elongation of a muscle). This variable follows an attractor dynamics, Equation (9) with an attractor defined by the position of the activity peak in the DNF, *u*(*x, t*). This attractor is only turned on when an activity peak is present in the motor DNF. The typical choice for λ(*x*) is λ(*x*) = *cx*, but generally, this factor is subject to a learning (gain adaptation) process (see Section 2.6.3).

In a WTA architecture, the motor variable ϕ [Equation (9)] may be implemented as a neural population without lateral connections, which receives input from the a motor WTA [that is analogous to the motor DNF in Equation(8)] through a set of synaptic connections, λ(*x*). This input is summed by the motor variable population. The critical difference of this dynamics to the DNF (or WTA) dynamics is that the motor command is defined by the activity of the population rather than the location of an activity peak in the population (Bicho et al., 2000).

### 2.5. Autonomy and cognitive control in DFT

Critically, in order to close the behavioral loop, the cognitive control of the neural architecture is necessary. In particular, the agent that has access to several perceptual and motor modalities has to decide at each point in time, which perceptual input to use to control the motor system and which effector of the motor system to use to achieve a behavioral goal. This problem was addressed recently in DFT in terms of modeling executive control in human cognition (Buss and Spencer, 2012) and in the behavioral organization in robotics (Richter et al., 2012).

The crucial element that gives a neural architecture the desired autonomy of executive control is based on the principle of intentionality (Searle, 1983; Sandamirskaya and Schoner, 2010a). In practice, this principle amounts to a structural extension of DNFs, so that every behavioral state of the system has two components—a representation of an intention, which eventually drives the motor system of the agent, and a representation of the condition-of-satisfaction (CoS), which is activated by the sensory input when the action is finished and which inhibits the respective intention. The CoS DNF is biased, or preshaped, by the intention DNF to be sensitive to particular sensory input, characteristics for the action outcome. This coupling from the intention to the CoS DNF carries a predictive component of the intentional behavior, which may be shaped in a learning process (Luciw et al., 2013). Together, the intention and the CoS comprise an elementary behavior (EB, Richter et al., 2012), which generally has the dynamics of Equations (10).

(10) $$\begin{array}{l} \tau {\dot{u}}_{int}(x,t)=-u_{int}(x,t)+h+\int f(u_{int}(x^{\prime },t))\omega (x-x^{\prime })dx^{\prime }\\ \;+S_{1}(x,t)-c_{1}\int f(u_{CoS}(y,t))dy, \end{array}$$

$$\begin{array}{l} \tau {\dot{u}}_{CoS}(y,t)=-u_{CoS}(y,t)+h+\int f(u_{CoS}(y^{\prime },t))\omega (y-y^{\prime })dy^{\prime }\\ \;+S_{2}(y,t)+c_{2}W(x,y)f(u_{int}(x,t)) \end{array}$$

Here, *u*_*int*_(*x, t*) is a DNF which represents possible intentions of the agent. These intentions may be motor or perceptual goals, which the agent aims to achieve through contact with the environment. For instance, “locate a red object” is a typical perceptual intention, “turn 30 degrees to the left” is an example of a motor intention. *x* is a perceptual or motor variable, which characterizes the particular intention; *S*_1_(*x, t*) is an external input which activates the intention. This input may be sensory (condition of initiation) or motivational (task input) (Sandamirskaya et al., 2011). *u*_*CoS*_(*y, t*) is the condition-of-satisfaction DNF, which receives a localized input from the intention DNF through a neuronal mapping *W*(*x, y*) (as introduced in Section 2.3). This input makes the CoS DNF sensitive to a particular part of the sensory input, *S*_2_(*y, t*), which is characteristic for the termination conditions of the intended perceptual or motor act. The mapping *W*(*x, y*) may be learned (Luciw et al., 2013). When the CoS DNF is activated, it inhibits the intention DNF by shifting its resting level below the threshold of the forgetting instability.

The DNF structure of an elementary behavior (EB) further stabilizes the behavioral state of the neural system. Thus, the intentional state of the system is kept active as long as needed to achieve the behavioral goal. The CoS autonomously detects that the intended action is successfully accomplished and inhibits the intention of the EB. Extinction of the previously stabilized intention gives way to the next EB to be activated. With this dynamics, the exact duration of an upcoming action does not need to be represented in advance (and action durations may vary to a large degree in real-world environments). The intentional state will be kept active until the CoS signals that the motor action has reached its goal. This neural-dynamic mechanism of intentionality enables autonomous activation and deactivation of different modalities of a larger neuronal architecture.

Since the intention and the CoS are interconnected DNFs, their WTA implementation may be achieved as described in Section 2.3.

### 2.6. Learning in DFT

The following learning mechanisms are available in the DFT framework.

#### 2.6.1. Memory trace of previous activity

The most basic learning mechanism in DFT is the memory trace formation, also called preshape. The memory trace changes the subsequent dynamics of a DNF and thus is considered an elementary form of learning. In neural terms, the memory trace amounts to local increase in excitability of neurons, which may be counterbalanced with homeostatic processes.

Formally, the preshape is an additional layer over the same dimensions as the associated DNF. The preshape layer receives input from the DNF, which is integrated into the preshape dynamics as an attractor that is approached with a time-constant τ_*l*_/λ_*build*_, Equation (11). This build-up constant is slower than the time-constant of the DNF dynamics. When there is no activity in the DNF, the preshape decays with an even slower time-constant, τ_*l*_/λ_*decay*_ in Equation (11).

(11) $$\begin{array}{l} \tau_{l}\dot{P}(x,t)=\lambda_{build}(-P(x,t)+f(u(x,t)))f(u(x,t))\\ \;-\lambda_{decay}P(x,t)(1-f(u(x,t))). \end{array}$$

Here, *P*(*x, t*) is the strength of the memory trace at site *x* of the DNF with activity *u*(*x, t*) and output *f*(*u*(*x, t*)), λ_*build*_ and λ_*decay*_ are the rates of build-up and decay of the memory trace. The build-up of the memory trace is active on sites with a high positive output *f*(*u*(*x, t*)), the decay is active on the sites with a low output. The memory trace *P*(*x, t*) is an additive input to the DNF dynamics.

The memory trace formation can be used to account for one-shot learning of object categories (Faubel and Schöner, 2009), representation of visual scenes (Zibner et al., 2011), or action sequences (Sandamirskaya and Schoner, 2010b).

In a neuromorphic WTA implementation, the memory trace, or preshape, may be interpreted as the strength of synaptic connections from the DNF (or WTA), *u*(*x, t*), to a “memory” population. This “memory” population activates the preshape by transmitting its activation through the learned synaptic connections, *P*(*x, t*). Learning of the synaptic connections amounts to attractor dynamics [as in the first parenthesis of Equation (11)], in which the pattern of synaptic connections approaches the pattern of the DNF's (WTA's) output. This learning dynamics may also be implemented as a simple Hebbian rule: the synaptic weights which connect active sites of the DNF (WTA) with the memory population are strengthened. Another possible interpretation of the preshape as a change in the resting levels of individual nodes in the DNF (WTA) is harder to implement in neuromorphic WTA networks.

#### 2.6.2. Learning mappings and associations

When the memory trace dynamics is defined within a structure with a higher dimensionality than the involved DNFs, the preshape dynamics leads to learning of mappings and associations. The dynamics of an associating map is similar to the memory trace dynamics, Equation (12).

(12) $$\tau \dot{W}(x,y,t)=\epsilon (t)(-W(x,y,t)+f(u_{1}(x,t))\times f(u_{2}(y,t))).$$

The weights function, W(x, y, t), which couples the DNFs *u*_1_(*x, t*) and *u*_2_(*y, t*) in Equation (12), as well as in Equations (4, 5), has an attractor at the intersection between positive outputs of the DNFs *u*_1_ and *u*_2_. The intersection is computed as a sum between the output of *u*_1_, expanded along the dimensions of the *u*_2_, and the output of the *u*_2_, expanded in the dimensions of the *u*_1_, augmented with a sigmoidal threshold function (this neural-dynamic operation is denoted by the × symbol). The shunting term ϵ(*t*) limits learning to time intervals when a rewarding situation is perceived, as exemplified in the architecture in Section 3.

This learning mechanism is equivalent to a (reward-gated) Hebbian learning rule: the cites of the DNFs *u*_1_ and *u*_2_ become coupled more strongly if they happen to be active simultaneously when learning is facilitated by the (rewarding) signal ϵ(*t*). Through the DNF dynamics, which builds localized activity peaks in the functionally relevant states, the learning dynamics has the properties of the adaptive resonance networks (ART, Carpenter et al., 1991), which emphasize the need for localization of the learning processes in time and in space.

#### 2.6.3. Adaptation

Adaptation [Equation (13)] is considered a learning process, which amounts to an unnormalized change of the coupling weights (gains) in a desired direction. A typical example is learning in the transition from the DNF's space-code to the rate-code of motor dynamics.

(13) $$\begin{array}{l} \tau \dot{\lambda }(x,t)=\epsilon (t)f(u(x,t))\\ \;\epsilon (t)=\text{error}\times \text{time window} \end{array}$$

Here, λ(*x, t*) is a matrix of weights, or gains, defined over the dimension of the DNF, *u*(*x, t*), which is coupled to the motor dynamics, as in Equation (9). The gain changes in proportion to the output of the driving DNF, *u*(*x, t*), in a learning window, defined by the term ϵ(*t*). The learning window is non-zero in a short time window when an intended action within EB, to which the DNF *u*(*x, t*) belongs, is finished (the respective *u*_*CoS*_ is active), but activity in the intention DNF is not yet extinguished. The *error* is determined in a DNF system, which compares the outcome of an action with the intended value of the motor variable and determines the direction of change of the weights in λ(*x, t*).

Now that all neural-dynamic structures developed within DFT are presented, which may be implemented in hardware neuronal networks through the WTA architecture, I will introduce an exemplar robotic architecture, which integrates these mechanisms in a neural-dynamic system, which generates behavior and learns autonomously.

## 3. An example of an adaptive architecture in DFT

### 3.1. The scenario and setup

The simple, but functioning in a closed loop learning architecture presented in this section employs several of the principles, presented above, such as categorization properties of DNFs, coupling between DNFs of different dimensionality, coupling to sensors and motors, autonomous action initiation and termination, as well as learning.

The robot, used to demonstrate the closed-loop behavior of a neuromorohic agent, consists of an eDVS camera and a pan-tilt unit. The eDVS camera has 128x128 event-based pixels, each sending a signal when a luminance change is detected. The pan-tilt unit consists of two servo motors, which take position signals in the range 0–2000 and are controlled to take the corresponding pose with a small latency. The task for this robot is to direct its gaze at a small blinking circle, which is moved around on a computer screen in front of the robot. A successful looking movement leads to the blinking circle settled in the central portion of the robot's camera array.

In order to accomplish this task, the robot, similarly to an animal, needs to detect the target in the visual input and in particular, estimate and represent its location relative to the center of the field of view of the robot. Next, according to the current location of the target, the system needs to select a motor command, which will bring the target into the center of the field of view. Thus, the system needs to select the desired values for pan and tilt, which will be sent to the servo motors.

This simple task embraces the following fundamental problems. First, the mapping between the target location and the required motor command is a priori unknown. The system needs to calibrate itself autonomously. In particular, the system needs to learn a mapping between the position of the input in the camera array and the motor command, which will bring the target in the center of the visual field. The second fundamental problem revealed in this setting is that when the camera moves, the perceived location of the target on the surface of the sensor changes, and the system needs a mechanism to keep the initial location of the target in memory in order to learn the mapping between the visually perceived locations and the motor commands. The third problem is the autonomy of the looking behavior: the systems needs a mechanism to update the target representation after both successful and unsuccessful looking actions.

**Figure 4:**
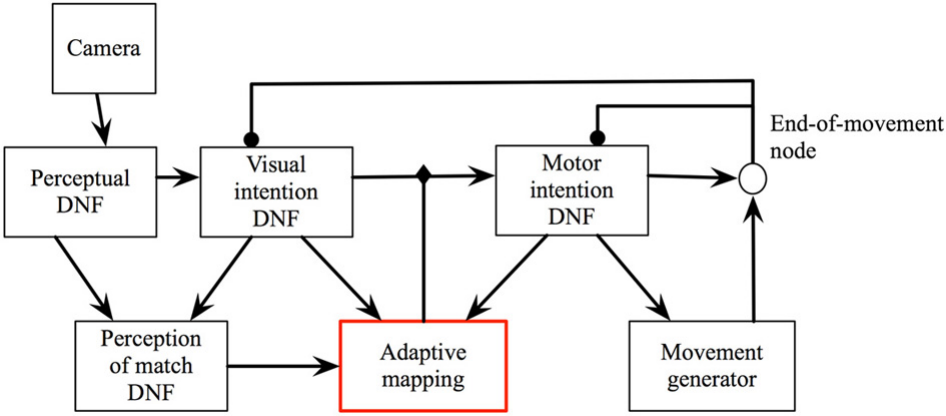
**The DFT architecture for looking**. See main text for details.

Figure 4 shows the scheme of the DNF architecture, which demonstrates how all these problems may be addressed in a closed-loop system. Next, I will present the dynamical structures, which constitute the architecture.

### 3.2. The neural-dynamics architecture

#### 3.2.1. Perceptual DNF

The perceptual DNF is coupled to the eDVS, as described in Section 2.2 and effectively performs a low-pass filter operation on the camera input in time and in space. This DNF builds peaks of activation at locations, where events are concentrated in time and in space in the visual array of the robot.

#### 3.2.2. Visual intention DNF

**Figure 5:**
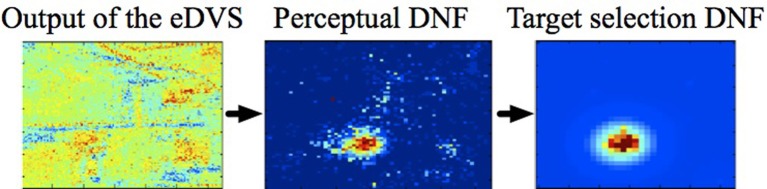
**The cascade from the visual input to perceptual DNF to the visual intention (target) DNF segregates and stabilizes the selected region in the input stream**.

This DNF builds sustained activity peaks that represent the target locations (Figure 5). The peaks are sustained even if the input, which initiated them ceases or moves. Thus, even during or after a gaze movement, the representation of the current target is stably represented. This allows, on the one hand, the robust coupling to the motor system (the attractor, set for the motor system, is guaranteed to be kept constant for the time of the movement). On the other hand, this memory system enables learning, since the representation of the previous target is still active when a rewarding input is perceived after a successful gaze.

#### 3.2.3. Motor intention DNF

The visual intention DNF represents the target of the current gaze action in sensory, here visual, coordinates. The movement generation system takes attractors in the motor coordinates, however (here, the desired pan and tilt). The motor intention DNF is defined over the motor coordinates and an activity peak in this DNF creates an attractor for the motor dynamics and initiates a gaze movement.

#### 3.2.4. Condition of satisfaction node

The CoS DNF in this architecture is a zero-dimensional CoS node, since it monitors directly the state of the motor system, which is characterized by two single-valued variables, pan and tilt. The CoS node is activated when the motor action is accomplished [Equation (14)].

(14) $$\tau {\dot{v}}_{cos}(t)=-v_{cos}(t)+h+c_{exc}f(v_{cos}(t))+c\int f(u_{mot}(y,t))dy+c_{a}f_{diff},$$

where *v*_*cos*_(*t*) is the activation of the CoS node for either the pan or the tilt movement (the CoS of the overall movement is a thresholded sum of the two CoSs). The CoS node is activated if (1) there is activity in the motor intention DNF, *u*_*mot*_, and (2) the detector *f*_*diff*_ = *f*(0.5 − |ξ_*pan*_ − *pȧn*|) signals that the state variable for the pan or the tilt dynamics reaches the respective attractor, ξ. *c* and *c*_*a*_ are scaling constants for these two contributions, *c*_*exc*_ is the strength of self-excitation of the CoS node.

The activated CoS node inhibits both the motor and the visual intention DNFs below activation threshold. The otherwise self-sustained activity peaks in these DNFs cease, which causes the CoS node loose its activation as well. The intention DNFs are released from inhibition and regain their initial resting levels, allowing the sensory input to induce a stabilized representation of the next target.

#### 3.2.5. The transformation array

The transformation between the visual and the motor coordinates, needed to achieve a particular behavioral goal, e.g., center the target object in the visual field, is a priori unknown. In the DFT architecture presented here, this transformation is represented by a randomly initialized coupling matrix, which implements a potential all-to-all connectivity between the two DNFs. Thus, an active visual intention DNF initially induces a peak at a random location in the motor DNF. The lateral interactions in the motor DNF ensure that a peak may be built, although the connection matrix is random (and sparse) in the beginning of the learning process.

In the transformation array, a learning dynamics is implemented [Equation (12)]. The learning window, λ(*t*) is defined by the activity in the visual match DNF, which signals when the visual input falls onto the central part of the camera array.

#### 3.2.6. The visual match DNF

The visual match DNF receives a preshape in the center of the field when the visual intention DNF is active. This preshaping input is equivalent to an expectation to perceive the target in the visual field, which biases the visual match DNF to be sensitive to the respective sensory input. The connectivity which enables this predicting coupling is assumed to be given here, but could potentially emerge in a developmental process [e.g., similar to Luciw et al. (2013)].

(15) $$\begin{array}{l} \tau {\dot{u}}_{match}(x,t)=-u_{match}(x,t)+h+\int f(u_{match}(x^{\prime },t))w(x-x^{\prime })dx^{\prime }\\ \;+f(u_{perc}(x,t))+c_{G}(x,t)\int f(u_{vis}(x,t))dx, \end{array}$$

In Equation (15), the visual match DNF, *u*_*match*_(*x, t*) is defined over the same (visual, 2D here) coordinates as the perceptual DNF, *u*_*perc*_, and the visual intention DNF, *u*_*vis*_, and receives a one-to-one input from the perceptual DNF, as well as a Gaussian-shaped input, *c*_*G*_(*x, t*) if there's activity in the visual intention DNF. When the visual match DNF is active, it drives learning in the transformation array, according to Equations (16, 12).

(16) $$\epsilon (t)=\int f(u_{match}(x,t))dx.$$

### 3.3. The dynamics of the architecture

**Figure 6:**
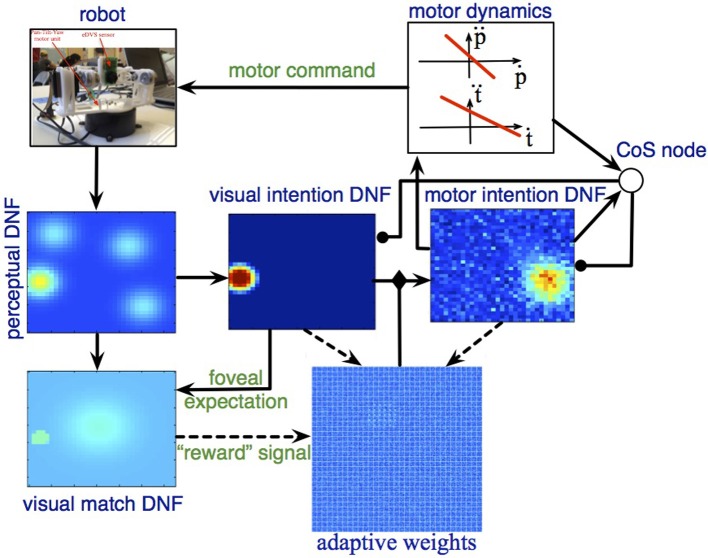
**The DFT architecture for autonomous looking and learning the sensorimotor map**. The robotic camera provides input to the perceptual DNF, which performs initial segregation of object-like regions in the visual stream. The visual intention DNF selects and stabilizes the spatial representation (in visual coordinates) of a single target for the upcoming looking action. Through adaptive weights, the visual intention DNF provides input to the motor intention DNF, which generates attractors for the motor dynamics. Motor dynamics signals completion of the looking act through the CoS node, which inhibits the intention DNFs. If the looking action brings the target object into the foveal (central) region of the field of view, the adaptive weights are updated according to the current (decaying) activation in the visual and motor intention DNFs.

Figure 6 show the DNF architecture for looking at work.

When salient visual input is perceived by the eDVS sensor, one or several activity peaks emerge in the perceptual DNF (Figure 6, left), the most salient of these peaks (i.e., the one that reached the activation threshold first) drives the visual intention DNF (Figure 6, middle) and induces a self-sustained activity peak in this DNF. The peak in the visual intention DNF is sustained even when the camera starts moving and the visual input shifts, representing the instantaneous goal of the upcoming camera movement.

The visual intention DNF induces an activity peak in the motor intention DNF through the coupling weights, which are random in the beginning of the learning process. A localized activity peak emerges in the motor intention DNF, formed by the lateral interactions in this field. The motor intention peak sets an attractor for the dynamics of the pan and the tilt control variables, which drive the robotic pan-tilt unit. When the control variables are close to the attractor, the CoS node is activated and inhibits the visual and the motor intention DNFs. Activity in the motor intention DNF ceases in a forgetting instability, which leads to the CoS node to loose its activation as well. The inhibitory influence on the intention DNFs is released and the visual intention DNF may build a new activity peak from the perceptual input.

When the camera movement is finished (event, detected by the CoS node), if the input falls onto the central part of the visual array, the visual match DNF is activated and triggers the learning process in the adaptive weights. In particular, the weights are strengthened between the currently active positions in the visual intention DNF and the currently active positions in the motor intention DNF, which correspond to the just-been-active intentions. When the CoS node inhibits the intention DNFs, learning stops and a new gazing action is initiated.

**Figure 7:**
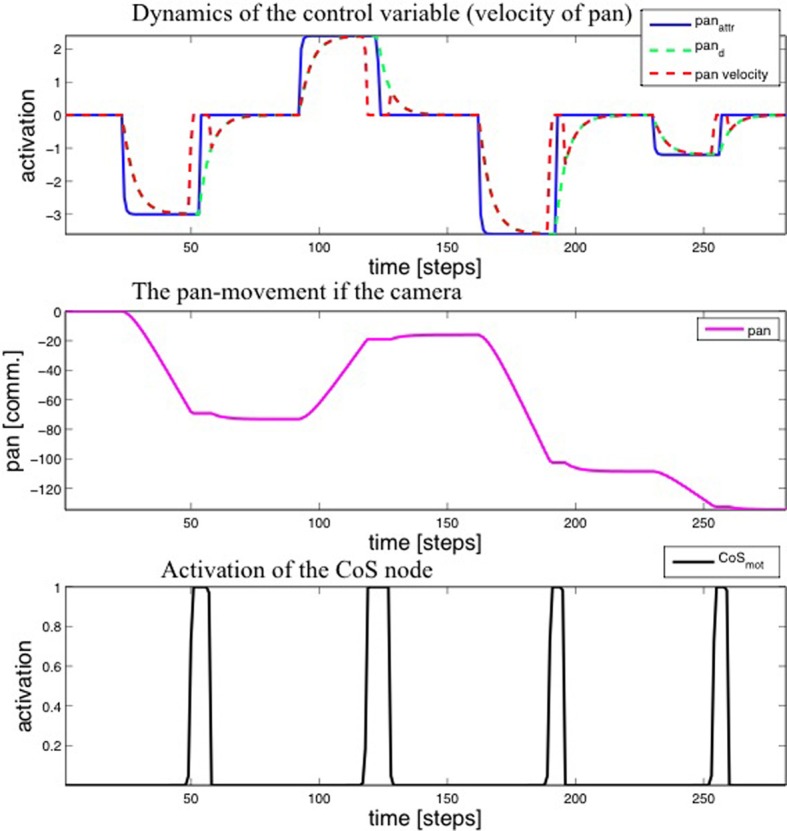
**Top:** Time-course of the activation of the motor variable (velocity of the pan joint) during four steps of the learning procedure. **Middle**: The value of the pan variable. **Bottom**: Activation of the CoS node.

**Figure 8:**
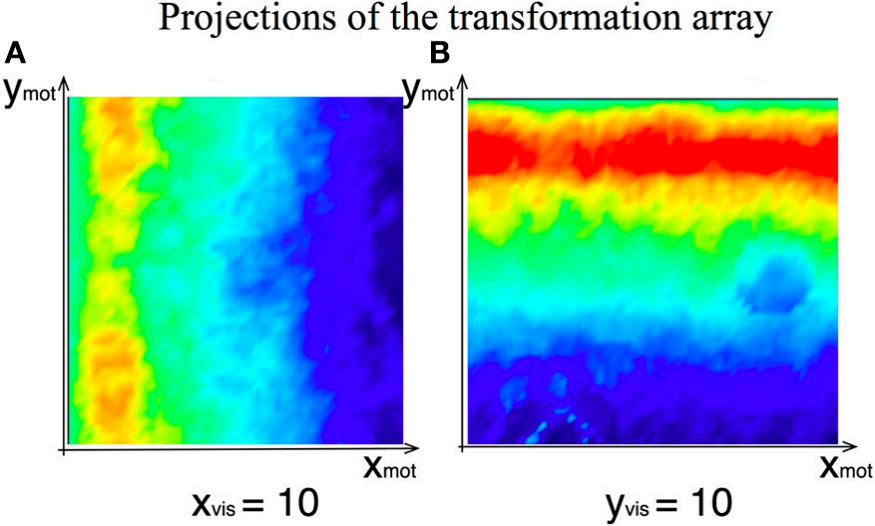
**Two exemplar projections of the learned 4D transformation array between the visual and the motor intention DNFs of the agent**. **(A)** Weights' strength at the given visual-intention DNF horizontal position as function of motor intention field coordinates (overlayed projections along *y*_*mot*_). **(B)** Weights' strength at the given visual-intention DNF vertical position.

Figure 7 shows the activity of the motor variables during the gaze movements in the learning process and Figure 8 shows the 2D projections of the 4D transformation matrix, learned over several hundred gaze movements to different target locations (Sandamirskaya and Conradt, 2013).

## 4. Discussion

### 4.1. General discussion

The principles of DFT presented in this paper set a possible roadmap for the development of neuromorphic architectures capable of cognitive behavior. As modeling framework, DFT is remarkable in its capacity to address issues of embodiment, autonomy, and learning using neural dynamics throughout. In this paper, I have reviewed the DFT mechanisms that provide for the creation of stabilized sensory representations, learned associations, coupled sensory-motor representations, intentionality, and autonomous behavior and learning. In an exemplar architecture, I demonstrated how the computational and architectural principles of DFT come together in a neural-dynamic architecture, that coupled a neuromorphic sensor to motors and autonomously generated looking behavior while learning in a closed behavioral loop. The categorization properties of DNFs achieve the stabilization of the visual input against sensory noise, while the memory mechanisms allow the relevant representations to be kept active long enough to parameterize and initiate motor actions and also drive the learning process after a successful movement. Adaptive couplings between DNFs together with a mechanism that enables autonomous activation and deactivation of intentions make for an architecture in which autonomous learning accompanies behavior.

In order to “translate” the language of behavior-based attractor dynamics of DFT to spiking networks implemented in VLSI, several possibilities have been reported recently. One solution (Neftci et al., 2013) constitutes a method to set parameters of the neuromorphic hardware in relation to parameters of a more abstract WTA layer. By measuring the activity of hardware units, the parameter mappings are calibrated in an automated procedure. Another way to translate DNF dynamics to spiking networks is to use the vector-encoding of a dynamical system in the neural-dynamic framework of Eliasmith (2005). This framework allows one to implement the attractor dynamics of DNFs in terms of a network of spiking units, which in its turn may define the parametrization for a VLSI neuromorphic network.

These powerful tools allow one to translate between levels of description and can be used to implement different models of cognition in order to facilitate the development of behaving, neuromorphic cognitive systems. DFT is one of the frameworks that defines the principles and constraints critical to this goal. There are of course several other frameworks that may be used for this purpose, each with its own advantages and limitations. Thus, the probabilistic framework allows one to use noisy and incomplete sensory information to infer hidden states of the environment and weigh alternative actions, which may bring the agent closer to its goals. Such a Bayesian framework has been applied both in the field of modeling human cognition [e.g., Griffiths et al. (2008)] and in robotics (Thrun et al., 2005). However, this framework has two limitations with respect to modeling human cognition. First, the probabilistic models focus on the functional or behavioral aspects of cognition and not the neuronal mechanisms underlying cognitive processing. They often require normalization procedures which are not trivial to implement neurally. Second, the probabilistic models often need an external mechanism to make inferences on the probability distributions and do not account for the process of decision making. Thus, the Bayesian architectures may achieve powerful performance and may be used to account for empirical data on human cognition, but they do not provide a process model of cognitive functions or offer a mechanism of how these functions are achieved or realized neurally. On the contrary, in neuronal modeling, the developed architectures are anchored in neuronal data and focus on the mechanisms and processes behind cognition. However, their functional implementations (i.e., embodiment) are typically limited and fail to address important problems such as representational coupling, autonomy, and development. DFT aims at bridging the two approaches to understanding cognitive processing—the functional (behavioral) and the mechanistic (neuronal)—and thus naturally fits the goal of providing for a tool to implement neuromorphic cognition. The scaling of DFT toward higher cognitive functions, such as concept representation, language, and complex action sequencing is currently under way.

This paper aims to reveal the formalized DFT principles and concepts developed in embodied cognition and autonomous robotics in such a way that they may be integrated into the language of spiking neural networks in VLSI hardware through the structure of WTA networks. DNF may be considered a functional description of the soft WTA networks. The successful implementation of soft WTA networks in VLSI devices to date opens the way to employing the architectural elements of DFT in spiking hardware architectures. These structural elements as summarized here are (1) coupling between fields of different dimensionality, (2) coupling to sensors through space-coding, (3) coupling to rate-coded motor dynamics, (4) application of principles of autonomy (intentionality), and (5) autonomous neural-dynamic learning. Some of the DFT principles, such as categorization and memory formation, are already probed in VLSI WTA networks, resulting in a framework of state-based computing in spiking networks. In addition, this paper formalizes mechanisms that allow for autonomous transition between stable states through the introduction of elementary behavior structures, namely the intention and the conditions-of-satisfaction. This formalization also enables autonomous learning and the robust coupling of WTAs to each other, to sensors, and to motor dynamics.

The DFT approach considers cognitive systems from a behavioral perspective while neuromorphic hardware system development aims at understanding the neuronal mechanisms underlying cognition. The fact that these two approaches converge to a mathematically equivalent object—a DNF or a soft WTA—as an elementary computational unit in the development of cognitive neuromorphic systems is a strong argument for the fundamental character of this computational element. Here, I aimed at establishing a common ground for future collaborative projects that can facilitate progress in both fields. The VLSI networks could scale up to produce cognitive autonomous behavior and the DFT framework could gain access to a neural implementation which is not only more efficient and biologically grounded, but also open to empirical links between the behavioral and neuronal dynamics. Bringing principles of DFT onto VLSI chips will, on the one hand, allow one to model human cognition and make predictions under both neuronal and behavioral constraints. On the other hand, the cooperation between the two fields could foster the development of powerful technical cognitive systems based on a parallel, low-power implementation with VLSI.

### Conflict of interest statement

The author declares that the research was conducted in the absence of any commercial or financial relationships that could be construed as a potential conflict of interest.
